# Paraspinal muscles density: a marker for degenerative lumbar spinal stenosis?

**DOI:** 10.1186/s12891-016-1282-6

**Published:** 2016-10-10

**Authors:** Janan Abbas, Viviane Slon, Hila May, Nathan Peled, Israel Hershkovitz, Kamal Hamoud

**Affiliations:** 1 Department of Anatomy and Anthropology, Sackler Faculty of Medicine, Tel Aviv University, Tel Aviv, Israel; 2 Department of Physical Therapy, Zefat Academic College, Zefat, Israel; 3 Department of Radiology, Carmel Medical Center, Haifa, Israel; 4 Faculty of Medicine in the Galilee, Bar-Ilan University, Zefat, Israel; 5 Department of Orthopaedic Surgery, The Baruch Padeh Poriya Medical Center, Tiberias, Israel

**Keywords:** Degenerative lumbar spinal stenosis, Paraspinal muscles density, Muscle cross-sectional area size, Computerized tomography (CT)

## Abstract

**Background:**

The condition of paraspinal muscles is known to be associated with some variables such as age, gender, and low back pain. It is generally agreed that these muscles play an important role in the stability and functional movements of the lumbar vertebral column. Although spinal instability has been shown to play an essential role in degenerative lumbar spinal stenosis (DLSS), the role of paraspinal muscles remains elusive. The main purpose of this study was to shed light on the relationship between the condition of paraspinal muscles and symptomatic DLSS.

**Methods:**

Two sample populations were studied. The first included 165 individuals with DLSS (age range: 40–88, sex ratio: 80 M/85 F) and the second 180 individuals without spinal stenosis related symptoms and low back pain (age range: 40–99, sex ratio: 90 M/90 F). Measurements were taken at the middle part of L3 vertebral body, using CT axial images (Philips Brilliance 64). Muscles density was measured in Hounsfield units (HU) using a 50 mm^2^ circle of the muscle mass at three different locations and the mean density was then calculated. The cross-sectional area (CSA) was also measured using the quantitative CT angiography method. Analysis of Covariance (adjusted for body mass index and age) was performed in order to determine the relationship between the condition of paraspinal muscles and symptomatic DLSS.

**Results:**

Individuals in the stenosis group had higher muscle density as compared to the control group. The CSA values for the erector spinae (both sexes) and psoas (males) muscles were significantly greater in the stenosis group as compared to their counterparts in the control group. Additionally, density of multifidus (both sexes) and erector spinae (males) muscles was significantly associated with symptomatic DLSS.

**Conclusions:**

Our results show that individuals with symptomatic DLSS manifest greater paraspinal muscles density and CSA (erector spinae), compared to the control group. Density of multifidus increases the likelihood of symptomatic DLSS.

## Background

The density of the paraspinal muscles and their cross-sectional area (CSA) size are known to be associated with variables such as age, gender and weight [1–6]. Current evidence suggests that these muscles are smaller in patients with chronic low back pain as compared to healthy individuals of a similar age [3, 5, 7]. It is generally agreed that muscles’ CSA and density reflect muscle performance and physical function [8–10] of individuals. Condition of muscle such as density, CSA size and fatty infiltration can be attained via medical imaging techniques that provide non-invasive and reproducible information [4, 11]. Computed tomography (CT) and magnetic resonance imaging (MRI) have been used for measuring CSA and the degeneration rate of muscles in patients with muscular dystrophy [12, 13].

The paraspinal muscles play an important role in the stability and functional movements of the lumbar vertebral column [14, 15]. Previous in vitro studies have reported that the lumbar spine is inherently unstable [16] and that much of its stability is dependent on the integrated function of the active, passive and neural sub-systems [17, 18]. Degenerative lumbar spinal stenosis (DLSS) is a clinical entity, an age-dependent phenomenon that is associated with degenerative changes of the three-joint complex and ligament flavum hypertrophy [19–21]. Although spinal instability has been shown to play an essential role in DLSS [22], the role of paraspinal muscles remains elusive.

The aim of this study was to examine via medical imaging techniques whether an association between the condition of paraspinal muscles and symptomatic DLSS exists.

## Methods

### Study groups

This study was conducted as a cross-sectional study and two groups of people were studied: the first, a control group, included 180 individuals without spinal-stenosis-related symptoms (age range: 40–99 years, sex ratio: 90 M/90 F). These subjects referred to the Department of Radiology from 2008 to 2010 for abdominal CT scans due to renal colic symptoms were interviewed. Individuals free of spinal stenosis symptoms and low back pain were included in the control group. The second, the DLSS group, included 165 individuals (age range: 40–88 years; sex ratio: 80 M/85 F), who were consecutively enrolled from 2006 to 2010 for a general study on etiology and pathophysiology of spinal stenosis [21, 23–25]. These subjects were referred to CT scans due to symptoms of spinal stenosis and were examined and interviewed by a spine surgeon post scanning. Inclusion criteria were presence of intermittent claudication accompanied by other symptoms related to spinal stenosis (low-back pain and radicular referred pain) [26–28], and reduced cross-sectional area of the dural sac (<100 mm^2^) [29–31] of at least one lumbar level (the radiological verification was carried out separately by two researchers). Individuals with congenital stenosis (AP of the bony canal < 12 mm) [26, 32], fractures, spondylolysis, tumors, Paget’s disease, steroid treatment, severe lumbar scoliosis (>20°) and iatrogenic (post laminectomy, post fusion) were not included in this group.

**Fig. 1:**
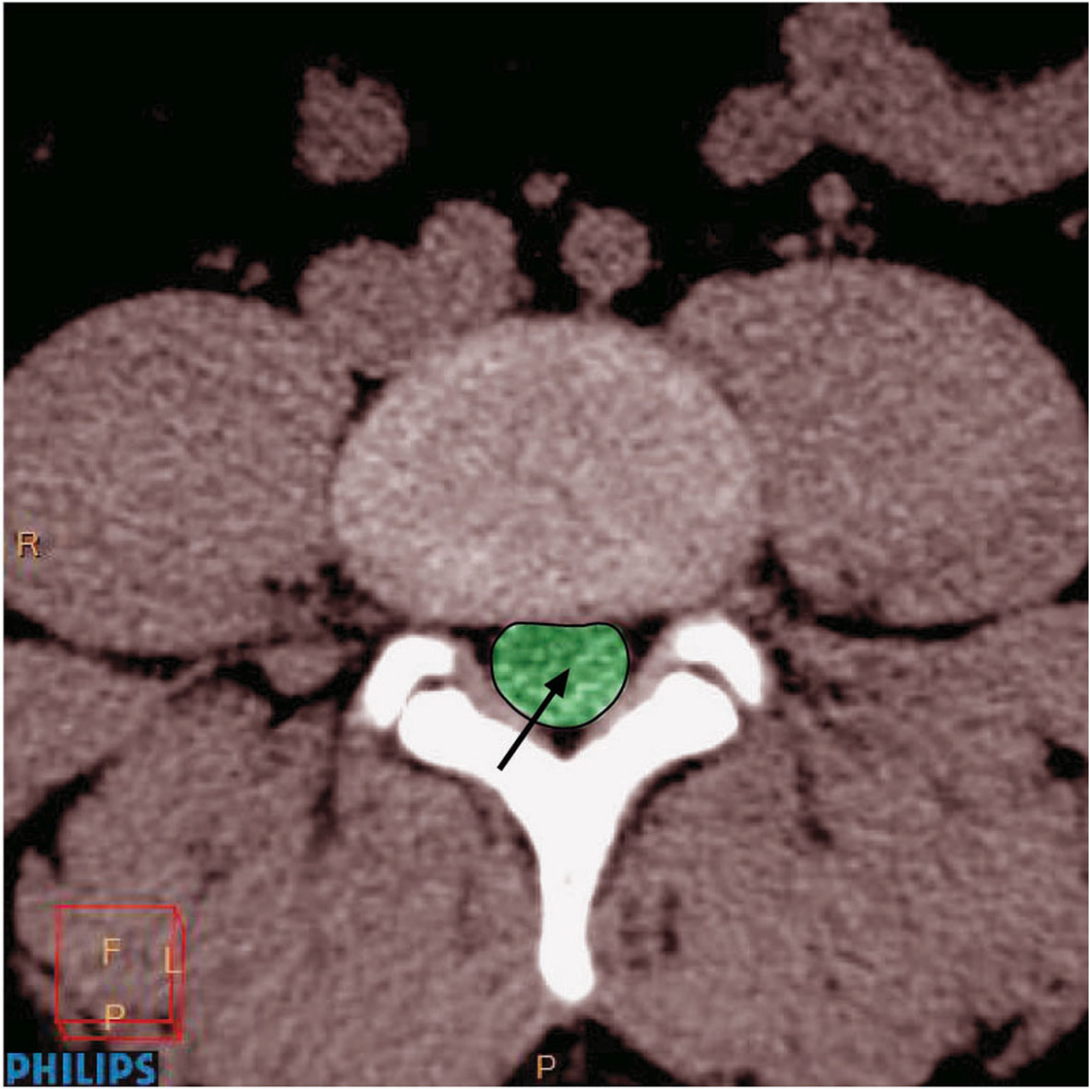
Measurement of lumbar cross-sectional area of the dural sac

The CSA of the dural sac was measured from CT scans (thicknesses of the sections were 1–3 mm and MAS, 80–250) in the axial plane at the lumbar intervertebral disc levels (Fig. 1), using Brilliance 64 Philips workstation (Medical System, Cleveland Ohio). This workstation enabled the processing of the scans in all planes and allowed a 3D reconstruction of the lower lumbar region. All CT images for both groups were taken in the supine position (extended knees) without contrast material.

The density and CSA of the paravertebral muscles were measured at the level of L3 mid- height since they are most extensive in this region [33, 34] and because the boundaries between the multifidus muscle and the erector spinae muscles become less clear as we descend caudally [9].

#### Evaluation of para-vertebral muscles

1. **Density of para-vertebral muscles** (psoas, multifidus and erector spinae) was measured in Hounsfield units (HU) on the left and right sides (Fig. 2). The value of each side (right, left) was obtained separately by calculating the mean density from measurements at three different locations (using a 50 mm^2^ circle), i.e., at the center of the muscle mass and at two additional points medial and lateral to it, close to the muscles’ margin. Densities of right and left muscles were then combined and the mean value was calculated.

**Fig. 2:**
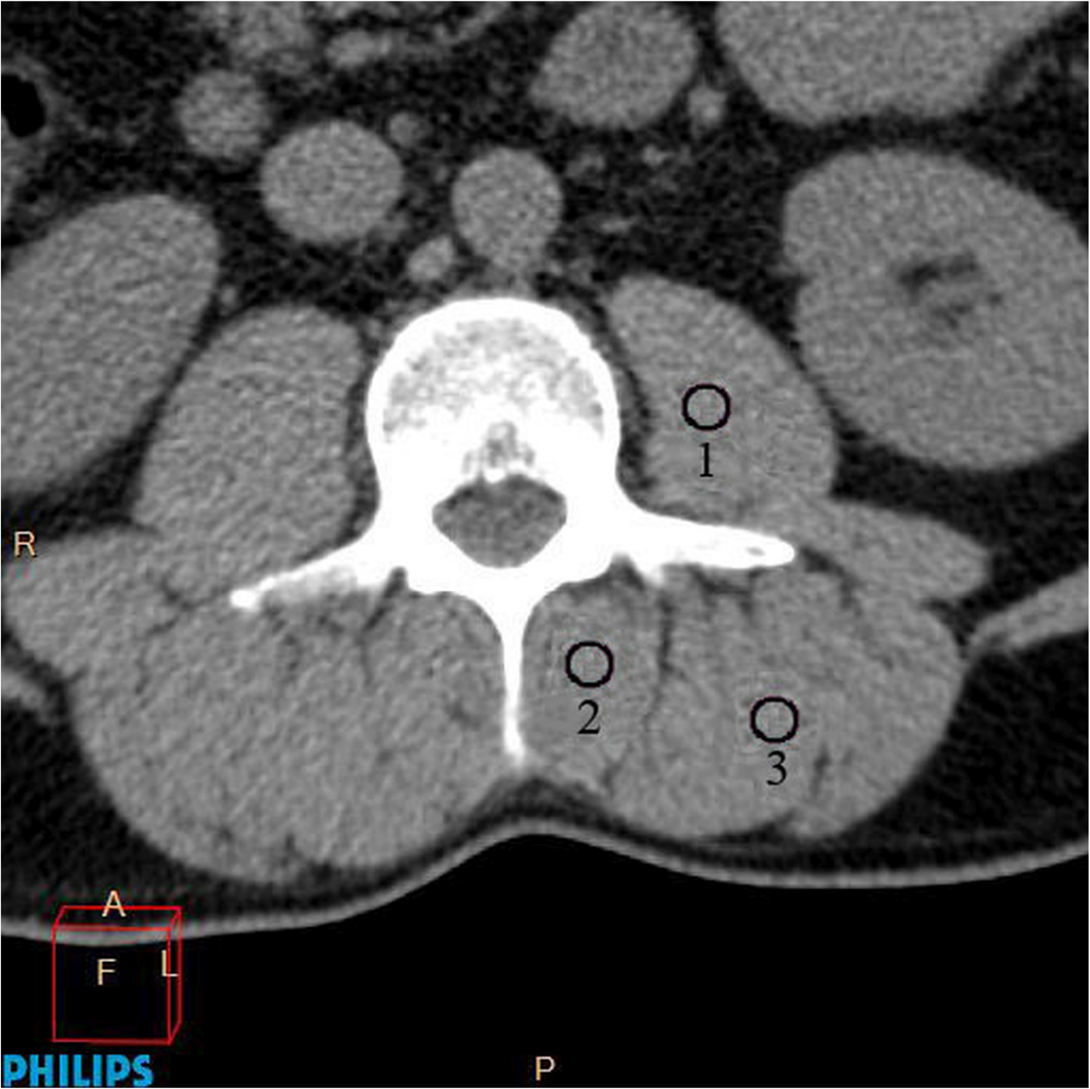
Measurement of para-vertebral muscle density: psoas (1), multifidus (2) and erector spinae muscles (3)
2. **Cross-sectional area (CSA) of the para-vertebral** muscles (psoas, multifidus and erector spinae) was measured on both sides separately and was defined by manually outlining the innermost fascial border surrounding the muscle, using the quantitative CT angiography method (Fig. 3). A threshold technique was then applied in order to eliminate the presence of other tissues such as fat and bones. The mean values for the right and left muscles were then calculated. This CSA measurement (excluding fat deposits) was proposed to be a better indicator of the muscle’s contractile ability than the total CSA [35–37].

**Fig. 3:**
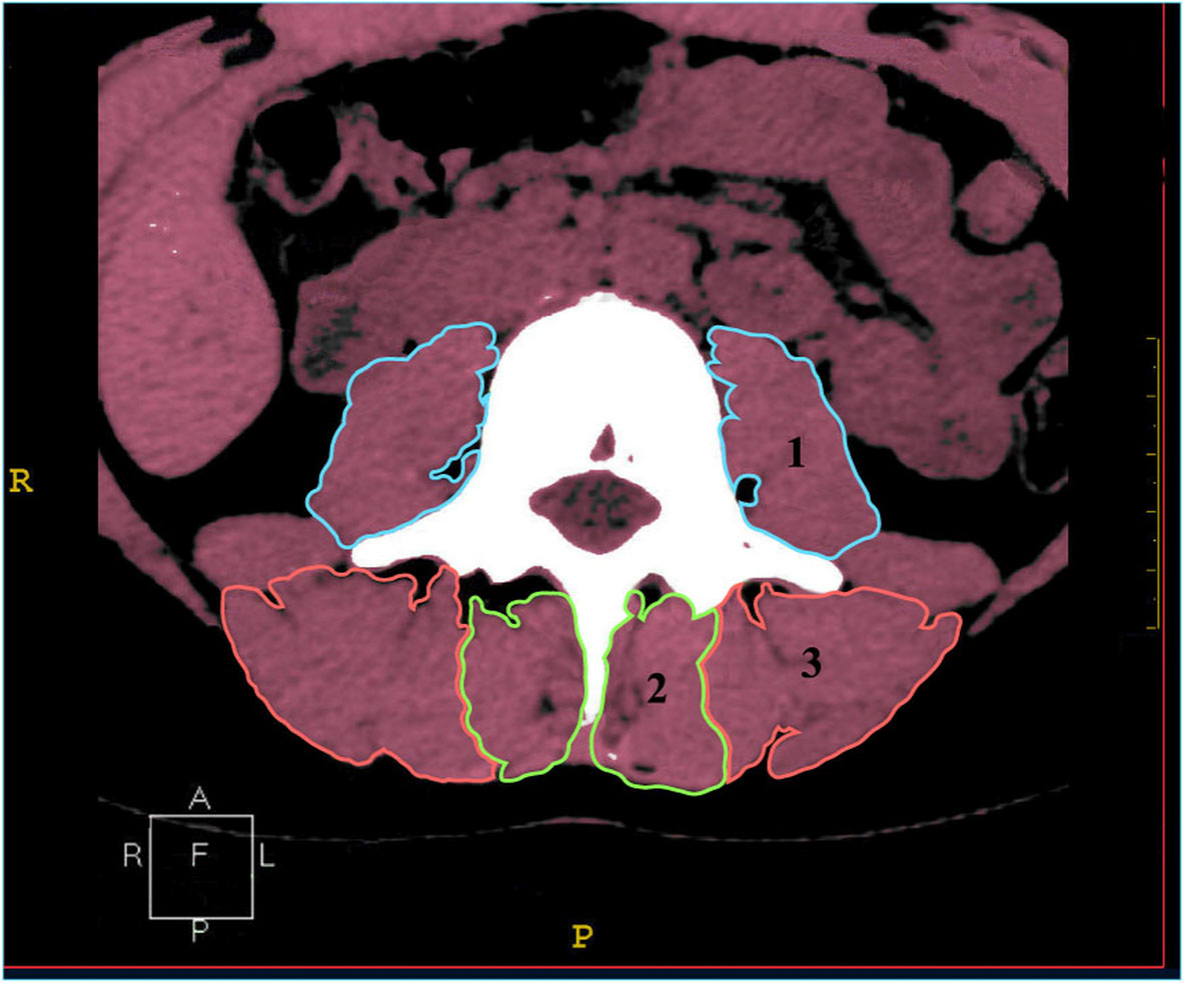
Measurement of cross-sectional area of psoas (1), multifidus (2) and erector spinae (3) muscles

#### Statistical analysis

The sample size of this study was based on the statistical power analysis (α = 0.05, β = 0.8).

Analysis of Covariance (ANCOVA) was performed for each gender separately in order to determine the association between the condition of paraspinal muscles and symptomatic DLSS (adjusted for body mass index and age). Logistic regression analysis was also carried out (separately for males and females) to reveal whether the paraspinal muscles increase the likelihood of symptomatic DLSS.

In order to determine the intra- and inter-tester reliabilities, intra-class correlations (ICCs) were calculated on repeated measurements for CSA and density of muscles and CSA of dural sac on 20 individuals. Intra-tester reliability was tested by one of the authors (JA) who carried out the measuring of the muscles two times, within intervals of 3–5 days. Inter-tester reliability involved two testers (JA and KH) who took the measurements using the same method within an hour of each other. Both testers were blinded to the results of the other. Significant difference was set at *P* < 0.0.5.

## Results

Both intra- and inter-tester reliability rates for muscle measurements were very high: the ICCs (intra-class correlation coefficient) obtained for the intra-tester tests were: CSA =0.940, density =0.920, and for the inter-tester tests: 0.922 and 0.90, respectively. In addition, the ICCs for both intra- and inter-tester reliability rates for CSA of the dural sac were very high: 0.930 and 0.890, respectively.

No significant difference was found in the mean age between the stenosis males and females and their counterpart in the control group (Table 1). However, both males and females in the stenosis group had greater BMI compared to the control.

**Table 1:** Mean age and body mass index (BMI) ± standard deviation (SD) of the control and the stenosis groups by gender. *N* = sample size

Study groups	Mean age (years) ± SD	Mean BMI (kg/m^2^) ± SD
Control males (*N* = 90)	62.9 ± 12.38	27.4 ± 4.21
Stenosis males (*N* = 80)	66.2 ± 10.82	28.9 ± 4.55
	*P* = 0.066	*P* = 0.021
Control females (*N* = 90)	62 ± 12.97	27.61 ± 5.13
Stenosis females (*N* = 85)	62.5 ± 8.63	31.48 ± 5.83
	*P* = 0.795	*P* < 0.001

### Para-spinal muscles and DLSS group

Both males and females in the stenosis group had higher (*P* < 0.05) muscle density as compared to their counterparts in the control group (adjusted for age and BMI) (Table 2). Additionally, it is noteworthy that the greatest density changes were found in multifidus muscle (22 to 24 %) rather than psoas or erector spinae (7–20 %) muscles.

**Table 2:** Mean density of paraspinal muscles (± SD) in the stenosis and control groups by sex

Sex	Muscle	Mean density (HU) ± SD		P value
		Control (*N* = 90)	DLSS (*N* = 80)	
Male	Psoas	40 ± 9	45 ± 9	<0.001
	Multifidus	34 ± 15	45 ± 12	<0.001
	Erector spinae	34 ± 11	43 ± 10	<0.001
		*N* = 90	*N* = 85	
Female	Psoas	40 ± 8	43 ± 10	0.01
	Multifidus	24 ± 16	31 ± 17	<0.001
	Erector spinae	29 ± 12	35 ± 15	<0.001

The CSA values for the erector spinae and psoas muscles were significantly greater in stenosis males as compared to their counterparts in the control group (adjusted for age and BMI). The mean CSA value for erector spinae muscle was 1793 mm^2^ and 1662 mm^2^ (stenosis males vs. control males) and for psoas 1097 mm^2^ and 1026 mm^2^, respectively. Yet, significant differences among females were noted only for the erector spinae muscle (1540 mm^2^ vs. 1345 mm^2^, *P* = 0.014) (Table 3).

**Table 3:** Mean cross sectional area (CSA) of paraspinal muscles (± SD) in the stenosis and control groups by sex

Sex	Muscle	Mean CSA (mm^2^) ± SD		P value
		Control (*N* = 90)	DLSS (*N* = 80)	
Male	Psoas	1026 ± 276	1097 ± 234	0.042
	Multifidus	589 ± 128	569 ± 111	0.331
	Erector spinae	1662 ± 394	1793 ± 369	0.011
		*N* = 90	*N* = 85	
Female	Psoas	628 ± 168	698 ± 145	0.076
	Multifidus	477 ± 117	524 ± 121	0.163
	Erector spinae	1345 ± 338	1540 ± 314	0.014

The results of the multivariable logistic analyses are presented in Table 4. Densities of multifidus for both gender (OR: 1.10–1.12; P ≤ 0.007) and erector spinae muscles in males (OR: 1.12; *P* = 0.039) were found to increase the likelihood of symptomatic DLSS development.

**Table 4:** Paraspinal muscles density that increases the likelihood for DLSS development for males and females, logistic regression analysis

Muscle	OR	(CI) 95 %	P value
Males			
Density of multifidus	1.12	1.023–1.165	0.007
Density of erector spinae	1.12	1.004–1.177	0.039
Female			
Density of multifidus	1.10	1.032–1.12	<0.001

*OR* odds ratio, *CI* confidence interval

## Discussion

The present study is, to the best of our knowledge, among the largest to examine the relationship between paraspinal muscles density and cross-sectional area (CSA) and symptomatic degenerative lumbar spinal stenosis (DLSS).

The results of our study show that both males and females in our stenotic group manifested significantly greater density of the paraspinal muscles and higher CSA (for the erector spinae muscle) at the L3 level compared to the control group. Additionally, the density of the multifidus (both sexes) and erector spinae (males) muscles was found to increase the likelihood of symptomatic DLSS. However, no point of discrimination could be established to differentiate between individuals with and without symptomatic DLSS.

Our findings are in contrast to the general view that paraspinal muscle density and size tend to show lower CSA and density in individuals with low back pain [36, 38, 39], disc pathology [40], degenerative lumbar flat back [41], degenerative kyphosis [42] and spinal stenosis [43]. Yarjanian and colleagues reported that CSA of paraspinal muscles at L5-S1 level had decreased in individuals with clinical stenosis (*n* = 15) compared with the asymptomatic group. The observed muscle atrophy was attributed to muscle denervation and/or disuse [43]. This result may seem to contradict ours as we have found the reverse phenomenon, but not so. Indeed individuals with lumbar spinal stenosis manifest evidence for paraspinal denervation [44–46], mainly at the L5 level [47] hence, muscle atrophy at L5-S1 level is expected. This results in inadequate lower lumbar segmental stabilization, which in turn requires compensation by the paraspinal muscles at higher levels. Increase in density of paraspinal muscles at L3 level and above in DLSS individuals is therefore anticipated. Support to the above notion can be found in Leinonen et al. [48] study which reported that individuals with clinical lumbar spinal stenosis manifested (“unexpectedly”) good back muscle endurance and significantly lower paraspinal muscle fatigue compared to healthy subjects. These authors claimed that the poor relationship between muscle endurance and denervation observed in individuals with clinical stenosis may indicate the compensatory use of other trunk extensor muscles.

The fact that degenerative lumbar spinal stenosis is triggered by segmental instability [22], explains why this disease is commonly accompanied by facet-joints arthrosis, intervertebral disc disease, ligamentum flavum hypertrophy and osteophyte formation [49, 50].

Instability was determined by Pope and Panjabi [51] as a mechanical entity that is related to loss of stiffness. The paraspinal muscles act to support the spine and maintain its stability [52]. Multifidus and erector spinae muscles also act as back extensors [53]. The psoas muscle is considered a major flexor for the hip joint, an intersegmental extensor in the mid lumbar region, and generally functions as an active postural muscle [54–56]. The different functions of the three muscles in stabilizing the lower spine explain why multifidus and erector spinae were more responsive to DLSS than the psoas.

Two parameters were used in the current study to evaluate muscle status: first, muscle density that reflects quantity of muscle fibers, as well as the area of a muscle’s fiber and the general packing of the contractile material [57]. Second, CSA, which is determined mainly by the total number of muscle fibers and, to a lesser degree, by the size of the fibers [58]. According to our study, density is the more sensitive parameter of the two. We here argue that during the initial phase of spinal stenosis cascade, when the spine segment is prone to repetitive high loading and shearing forces that affect its stiffness (stability), the paravertebral muscles may enhance activities and contractions in order to compensate for increased segmental mobility. Only at a later stage additional mechanisms such as the thickening of ligamentum flavum and hypertrophy of facet-joints are involved. Supporting studies have reported that in short-term spine instability, muscles can respond actively and reduce spinal movements [59, 60], whereas in the long term, tissue remodeling in the form of osteophyte formation and ligament hypertrophy may help to restore stability [22, 61]. It has also been claimed that the human spine responds to changes in stability by utilizing its own passive and active preventive mechanisms, i.e., muscles, ligaments and bone structures [59]. It has recently been shown [62] that patients with low-back pain (LBP) have significantly larger CSA of the psoas at the levels of L3-4 and L4-5 than the control group. The authors hypothesized that hypertrophy of the psoas was the result of its increased activity in maintaining stability of the lumbar spine.

Based on the above findings, we argue that the paraspinal muscles work to control mobility and to achieve stability in symptomatic DLSS individuals, thus resulting in thicker and denser muscles. This muscle behavior, essentially a recruitment strategy to compensate for reduced stability, was noted in other parts of the body, for example the trunk [63, 64]. Additionally, in vitro studies [16, 59] demonstrate that the multifidus muscle has the capacity to restore control of segmental motion following injury. Positive correlation between training programs and increased paraspinal muscle features (density and CSA) was also reported [65–68]. Some studies have also connected the condition of paraspinal muscles with physical activities [9, 10, 68] and spine-fusion [5, 7, 69]. It can be argued that the greater density and CSA of the paraspinal muscles in the DLSS group is due to hyperactivity (spasm) of the muscles to limit motion and control for back pain [70]. However, this hypothesis can be rejected as no association has been found between severity of low back pain and the CSA of paraspinal muscles [71]. Moreover, paraspinal muscles atrophy has been noted in patients with acute or subacute LBP compared to controls [4, 68].

Our results are not in agreement with those of Kalichman et al. who found no association between paraspinal muscles density and radiological spinal stenosis [6]. Conflicting data have also been presented by others with regard to degenerative lumbar spine (e.g., facet-joints arthrosis, disc space narrowing) [6, 41, 42] and have demonstrated an attenuation in paraspinal features. This may be attributed to the fact that different inclusion criteria were used for the study groups (e.g., clinical vs. radiological stenosis) and not all degenerated spinal segments necessarily develop instability [72].

Several studies have shown a negative correlation between the condition of paraspinal muscle and LBP and/or disc pathology [4, 36, 37, 39, 69, 73]. Others have disputed this and reported no correlation between the condition of the muscles and LBP [6, 10, 71, 74]. These conflicting data can perhaps be explained by one of the following: (a) since LBP disorder is recognized as a multi-factorial origin [75], muscle condition in these individuals can vary, (b) different methods were applied for measurements (e.g., MRI, CT and ultrasonography), (c) various measurements (e.g., density, total CSA and/or free-fat CSA) and different lumbar spinal levels and locations were used.

A correlation between symptomatic DLSS and paraspinal muscle density was noted in our study. Changes in CSA were less significant. A possible explanation for this (changes in densities not in CSA) is that under intense muscle activation, changes in density will precede changes in CSA [58, 76]. Furthermore, after training, the magnitude of changes in density will be 2–3 times greater than changes in CSA [77].

Our findings indicate that the greatest change in muscle density was notable for multifidus muscle (less for psoas and erector spinae); it was also a significant factor to increase the likelihood for symptomatic DLSS in both sexes. This result is not surprising as the multifidus is the main muscle controlling spinal motion and also contributes to nearly 2/3 of the stiffness at L4-5 [78].

Approximately one-third of the elderly population manifests radiological stenosis without symptoms [79, 80]. Therefore, diagnosis of clinical syndrome of spinal stenosis must be carried out based on the combination of symptoms and signs together with the imaging findings [81]. Others have also underscored the importance of history and physical examination in determining the clinical syndrome of LSS [82] and the caution that physician should take when evaluating older patients with suspected spinal stenosis [83].

Since the increased paraspinal density in symptomatic DLSS individuals was a specific radiological finding for this disorder and not related to other degenerative lumbar spine disease, we suggest that this measurement can be used as a radiological marker for detecting the clinical syndrome of DLSS. Nevertheless, to establish reliable standards for DLSS, a much larger sample is required.

### Study limitation

The outcomes of this study warrant further investigations and verifications to determine whether our results are reproducible on different lumbar spine levels (e.g., L1, L4 and L5 rather than L3), locations and populations. An MRI study may provide higher resolution and clearer images of the soft tissue; this technique may be preferable to CT. Finally, one might argue that our finding may be due to the negative effects of renal colic in the control population (as some of them may indeed suffer from short episodes of low back pain in the past) not the ‘positive’ effect of the stenosis. However, considerable changes in the mass of muscles in individuals with a short episode of low back rather than a chronic condition, are not expected.

## Conclusions

The current study shows that individuals with symptomatic DLSS manifest greater paraspinal muscles density and CSA (erector spinae) compared to the control group. Density of multifidus (both sexes) is significantly associated with symptomatic DLSS.

## Abbreviations

BMI: Body mass index
CI: Confidence interval
CSA: Cross-sectional area
CT: Computed tomography
DLSS: Degenerative lumbar spinal stenosis
ICCs: Intra-class correlations
LBP: Low back pain
MRI: Magnetic resonance imaging
OR: Odds ratio
SD: Standard deviation
